# Responsiveness to Tocilizumab in Anti-Acetylcholine Receptor-Positive Generalized Myasthenia Gravis

**DOI:** 10.14336/AD.2023.0528

**Published:** 2024-04-01

**Authors:** Dongmei Jia, Fenghe Zhang, Huining Li, Yi Shen, Zhao Jin, Fu-Dong Shi, Chao Zhang

**Affiliations:** ^1^Department of Neurology and Institute of Neuroimmunology, Tianjin Medical University General Hospital, Tianjin Medical University Tianjin, China.; ^2^Department of Neurology, Third Central Hospital of Tianjin, Tianjin, China.

**Keywords:** generalized myasthenia gravis, tocilizumab, treatment, QMG, improvement

## Abstract

Tocilizumab, a humanized IL-6R monoclonal antibody, has been used in autoimmune diseases closely related to humoral immunity. This report aims to evaluate the efficacy and safety in patients with anti-acetylcholine receptor-positive (AChR+) generalized myasthenia gravis (gMG). We performed a prospective, open-label, single-arm study in patients with gMG in a 48-week follow-up. All patients were AChR+ and were given tocilizumab by intravenous infusion at a dose of 8 mg/kg at intervals of 4 weeks. The primary endpoint was mean change from baseline in quantitative MG (QMG) score at week 12. The secondary endpoints were mean changes from baseline in MG activities of daily living (MG-ADL) score, AChR-ab titers, and the dosage of oral prednisone at week 12. At week 48, QMG, MG-ADL, and the use of prednisone were also evaluated. Fourteen gMG patients were enrolled and all of them completed the study. Tocilizumab treatment started 8 (4-192) months after the onset of gMG. During tocilizumab treatment, the QMG score was significantly decreased from 15.5 (interqualile range, 9-26) at baseline to 4 (0-9) at week 12 (*p* < 0.001). The change of ADL was decreased from 14.5(11-19) at baseline to 4 (0-19) at week 12 (*p* < 0.001) and the change of AChR-ab titers from 15 (7.5-19) at baseline to 6.8 (11.6-4.3) at week 12 (*p* < 0.001). The dosage of prednisone decreased from baseline 60 (20-65) mg/d to 30 (30-50) mg/d at week 12 (*p <* 0.001). By the end of the study, the QMG score was 2 (0-7) and MG-ADL score was 1.5 (0-6). 12 (85.7%) patients achieved minimal manifestations. 4 (28.6%) patients were able to discontinue prednisone. No patients experienced exacerbation at the end of the study. No serious adverse events were observed during follow-up. Tocilizumab treatment was associated with a good clinical response and safety over a 48-week observation period, as evidenced by significant improvements in QMG and MG-ADL.

## INTRODUCTION

Myasthenia gravis (MG) is an autoantibody-mediated disease of the neuromuscular junction and usually results in fluctuating fatigability and weakness of ocular, bulbar, and limb skeletal muscles. Over 80% of patients with MG have antibodies against the acetylcholine receptor (AChR-ab) that causes the compromise of the postsynaptic neuromuscular endplate. Most patients can be managed with acetylcholinesterase inhibitors glucocorticoids, and/or immunosuppressants. However, there are still some patients with MG who do not respond adequately or tolerate well to these treatments [1, 2]. Among them, generalized MG (gMG) patients may be at a high risk of frequent exacerbations and even myasthenic crises [3]. There is a practical need for better-tolerated treatments with clinical response for gMG.

Pathogenic AChR-ab could induce a significant increase of IL-6 in human muscle cells. ^4^ Sera IL-6 levels were upregulated in AChR-ab positive gMG patients and was closely associated with myasthenic disease activity [4, 5]. In experimental autoimmune MG (EAMG), AChR-ab may also contribute to the production of IL-6. After anti-IL-6 treatment, a significant improvement of clinical state was observed in a rat model [4, 6]. AChR-immunized IL-6 gene deficient mice had reduced germinal center formation and a decreased production of AChR-ab. The genetic evidence demonstrated a key role of IL-6 in the autoimmune response to AChR and in EAMG pathogenesis [7].

Tocilizumab, a humanized IL-6 receptor (IL-6R) monoclonal antibody, inhibits the binding of IL-6 to its receptor and reduces the pro-inflammatory activity of cytokines [3]. It is well known that tocilizumab can reduce the activation and proliferation of B cells and inhibit occurrence of inflammatory response and antibody generation [8]. Several randomized controlled trials have shown the effectiveness of tocilizumab in autoimmune diseases including rheumatoid diseases and neuromyelitis optica spectrum disorders [9, 10]. A previous report showed that tocilizumab treatment resulted in clinical improvement over several months in two rituximab-refractory gMG patients [11]. This suggested a possible efficacy of tocilizumab in patients with gMG. To further investigate the efficacy and safety of tocilizumab in gMG, we did a prospective study in a case series of fourteen patients.

## METHODS

### Study population

This study was approved by the Ethics Committee of Tianjin Medical University. The inclusion criteria included: 1) adult AChR (+) gMG patients; 2) patients who responded inadequately to steroids and at least one immunosuppressants (The inadequate response was defined when the patient met at least one of the following criteria: 1) QMG score or MG-ADL score improved by <25%; 2) the steroids dosage failed to reduce; 3) the MGFA post-intervention state (PIS) didn't improve)[12]; or 3) Patients do not have improvement in their QMG scores after treatment with intravenous immunoglobulin G (IVIg) or plasma exchange (PE), or need repeated IVIG or PE; or 4) patients who are intolerant to adverse effects of previous treatments. The exclusion criteria were the patients with: 1) age > 80 years old; 2) a history of thymectomy within 12 months prior to screening; 3) Myasthenia Gravis Foundation of America (MGFA) class I or MGFA Class V; 4) the use of rituximab within 6 months prior to screening; 5) the use of IVIg or PE within 4 weeks prior tocilizumab treatment; 6) frequent myasthenic crises. Informed consent was obtained from all patients in accordance with the Declaration of Helskinki II.

### Treatments and evaluation

Eligible patients received tocilizumab (Roche) treatment at a dose of 8 mg/kg every 4 weeks. Myasthenia Gravis Foundation of America (MGFA) types, AChR-ab titers, dosage of glucocorticoid and cholinesterase inhibitor, quantitative MG (QMG) score and MG activities of daily living (MG-ADL) score of the patients were recorded at baseline and at weeks 4, 8, 12, 16, 20, 24, 48 after the first tocilizumab infusion. AChR-ab titers were measured with enzyme-linked immunosorbent assay (ELISA) kit, United Kingdom RSR, RBA 324).

The primary endpoint was mean change from baseline in QMG score at week 12. The secondary endpoints were mean changes from baseline in MG-ADL score, AChR-ab titers, and the dosage of corticosteroid at week 12. All clinical evaluators underwent standardized training on the measurement of the primary and secondary outcomes, and patients were expected to be assessed by the same evaluator throughout the study.

### Statistical analysis

Continuous data were summarized by median (quantile) and qualitative data by frequency (%). The normality of continuous data was evaluated by the Kolmogorov-Smirnov test. Baseline and post-intervention outcomes compared by paired samples *t*-test or Wilcoxon’s signed rank test. Results in each post-intervention time were summarized by Bonferroni 95% confidence intervals (CIs) through error bars. The significant probability of two-tail tests was considered less than 0.05. Stata version 16 software (StataCrop LP, College Station, TX, USA) was used for statistical analysis. P value < 0.05 was considered statistically significant.

## RESULTS

### Demographics and baseline clinical characteristics

**Figure 1.**
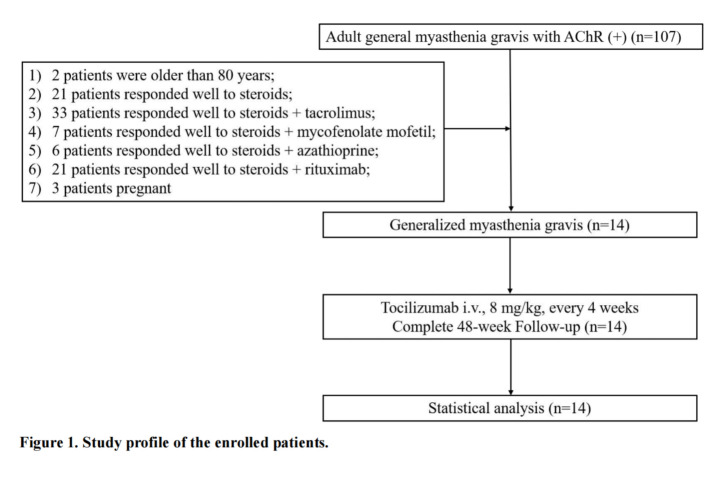
Study profile of the enrolled patients.

From November, 2019 to December, 2020, 107 AChR(+) gMG patients were screened and 14 of them were enrolled in the study (Fig. 1). 8 (57.1%) patients were female. The mean age was 59.6 ± 3.8 years. 9 (64.3%) of the patients had responded inadequately to prednisone and over 2 immunosuppressants (azathioprine, mycophenolate mofetil, tacrolimus or rituximab). 2 (14.3%) patients had thymoma and underwent thymectomy 2 years before the study. The mean time from onset of MG to initiation of tocilizumab treatment was 36.4 (15.7) months. The mean baseline dosage of prednisone is 60 (interqualile range, 20-65) mg/d. All the patients completed 48-week follow-up (Table 1).

**Table 1:** Baseline characteristics of patients.

Patient	Gender	Age	MGFA	Thymectomy	Medication history	Time from diagnosis to tocilizumab initiation (months)	Considerations for the switch to tocilizumab treatment	Pred dose before tocilizumab treatment (mg)	AChR-ab before tocilizumab treatment (nmol/L)
**1**	M	77	IIIa	-	Pred, AZA, MMF	6	Inadequate clinical response to Pred and MMF; Intolerance to AZA (severe liver dysfunction)	50	16.39
**2**	F	65	IIa	-	Pred, AZA, TAC	4	Intolerance to AZA (agranulocytosis) and TAC (moderate renal dysfunction)	40	15.5
**3**	M	60	IIIa	-	Pred, TAC, IVIG	120	Inadequate clinical response to Pred, TAC, IVIG	50	19.34
**4**	M	66	IIa	-	Pred, MMF, TAC	6	Inadequate clinical response to Pred and TAC; Intolerance to MMF (nausea and vomiting)	55	11.33
**5**	F	61	IIIa	-	Pred, AZA, MMF	5	Intolerance to AZA (severe liver dysfunction) and MMF (CD4+ T cells < 150 /μL and pneumonia)	60	17.43
**6**	F	77	IIIb	-	Pred, MMF, IVIG	4	Inadequate clinical response to Pred and IVIG; Intolerance to MMF (nausea and vomiting)	40	11.69
**7**	F	48	IIa	-	Pred, TAC, AZA	13	Inadequate clinical response to Pred, TAC; Intolerance to AZA (agranulocytosis)	55	15.67
**8**	F	38	IIa	-	Pred, AZA, IVIG	5	Intolerance to AZA (moderate liver dysfunction); Inadequate clinical response to Pred, IVIG	60	7.44
**9**	M	69	IIb	-	Pred, CYC, TAC	5	Intolerance to CYC (anorexia); Inadequate clinical response to Pred, IVIG	60	7.44
**10**	F	45	IIb	+	Pred, AZA, IVIG	192	Inadequate clinical response to Pred, AZA, IVIG	60	8.27
**11**	M	38	IV	+	Pred, MMF, IVIG	36	Inadequate clinical response to Pre, AZA, RTX, IVIG	60	16.5
**12**	F	43	IIb	-	Pred, MTX, TAC	10	Intolerance to MTX (severe leukopenia); Inadequate clinical response to Pred, TAC	60	16.46
**13**	F	80	IIIb	-	Pred, AZA, RTX, IVIG	120	Inadequate clinical response to Pre, AZA, RTX, IVIG	60	11.37
**14**	M	65	IIIa	-	Pred, MMF, RTX	9	Inadequate clinical response to Pred, MMF, RTX	60	15.4

Pred, prednisone; AZA, azathioprine; TAC, tacrolimus; MMF, mycophenolate mofetil; IVIG, intravenous immunoglobulin; MTX, methotrexate; CYC, cyclosporine; RTX, rituximab.

### Clinical outcomes

**Figure 2.**
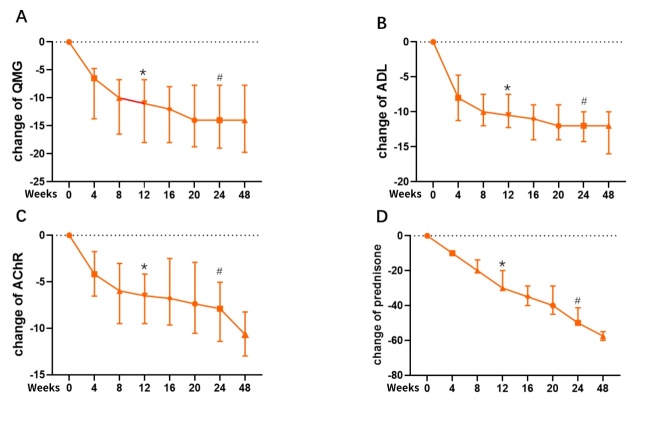
**The primary and secondary outcomes of the study**. The mean changes from baseline at different time points in quantitative myasthenia gravis (QMG) (A), myasthenia gravis activities of daily living (MG-ADL) (B), AChR-ab titers (C), and dosages of prednisone (D) were shown. ^*^Indicates the differences of the index at week 12 compared to baseline. # Indicates the differences of the index at week 24 compared to week 12. * p < 0.001. # p < 0.05.

The QMG scores were 15.5(interqualile range, 9-26) at baseline and decreased to 6(18-3) at week 4, 5 (9-3) at week 8, 4 (0-9) at week 12. From baseline to week 12, the score was significantly decreased (*p* < 0.0001) (week 24 vs. week 12, *p* = 0.0078; week 48 vs. week 12, *p* = 0.0039, respectively). The QMG score was 2 (0-9) at week 24 and 2 (0-7) at week 48, which was significantly decreased compared to week 12 (week 24 vs. week 12, *p* = 0.0078; week 48 vs. week 12, *p* = 0.0039, respectively). However, the difference in QMG was not statistically significant between week 24 and week 48 (*p* = 0.5000). The result showed a favorable effect from week 4, and the maximal efficacy reached a peak at week 24 (Fig. 2A).

For the secondary outcome, tocilizumab significantly reduced MG-ADL from a baseline of 14.5 (11-19) to 6(4-14) at week 4, 5.5 (2-6) at week 8, 4 (0-15) at week 12 (*p* < 0.0001). The change from baseline to week 12 was significantly (*p* < 0.0001). The MG-ADL score was 2 (0-15) at week 24 and 1.5(0-6) at week 48. The changes of MG-ADL were in line with those of QMG scores. The mean MG-ADL decreased at week 24 (*p* = 0.0195) and week 48 (*p* = 0.0049), compared to week 12 respectively. There was no significant difference between week 24 and week 48 (*p =* 0.1250) (Fig. 2B).

Tocilizumab also reduced titers of AChR-ab from 15(7.5-19) nmol/L at baseline, to 9.7 (4.8-11.5) nmol/L at week 4, 8.6(4.3-10.8) nmol/L at week 8, 6.8(11.6-4.3) nmol/L at week 12. The changes of AChR-ab titers paralleled with those of QMG score. There was a significant difference in the titers of AChR-ab between baseline and week 12 (*p* = 0.0001) (Fig. 2C). The AChR-ab titers were 5.6(3.7-9.3) nmol/L at week 24 and 3.5(0-6.9) at week 48. There was still a significant decrease from week 24 to week 48 (*p =* 0.0001) in AChR-ab titers (Fig. 2C).

The dosages of prednisone decreased significantly from 60 (20-65) mg/d at baseline to 30(30-50) mg/d at week 12, 10(0-45) mg/d at week 24, and 2.5(0-10) mg/d at week 44 (*p* < 0.001) (Fig. 2D). At week 24, two patients were able to stop prednisone. At week 48, 7 (50.0%) patients discontinued prednisone.

All patients completed the 48-week follow-up and13 patients had no further exacerbation of symptoms. Only one patient (Patient 10) in this study experienced myasthenic fluctuation during the follow-ups. She initially received tocilizumab treatment after 192 months of the onset of gMG. Her QMG decreased from 15 at baseline to 3 at week 12. At week 14, she received myomectomy because of myoma of uterus and her symptoms worsened after surgery. She had difficulty in breathing and raising her head and arms, with a QMG score 12. The patient was given 50 mg/d prednisone with oral pyridostigmine 360 mg per day and went on tocilizumab infusion. By the end of 48-week, prednisone was reduced to 35 mg/d and her QMG score was decreased to 9.

Till the end of the study, none of the patients developed an MG crisis and 12 (85.7%) patients achieved minimal manifestation status (MMS).

### Safety consideration

Adverse events were recorded among patients receiving tocilizumab treatment. Four patients (28.6%) had elevated sera alanine aminotransferase (ALT) and aspartate aminotransferase (AST) levels, which were 2-3 times higher than the upper limits of the normal range. After 2 weeks of oral tiopronin treatment, sera ALT and AST returned within the normal range. Two patients (14.3%) had upper respiratory tract infection with mild neutropenia. Symptomatic treatment alleviated the discomfort within two days. One female patient (7.1%) experienced two episodes of urinary tract infection with fever and oral cefuroxime treatment relieved her in a week. One patient (7.1%) experienced fatigue after the first infusion of tocilizumab, which subsided within five days. One patient (7.1%) had slightly increased sera cholesterol, and after 1 month of probucolum and acipimoxin treatment, sera cholesterol was decreased to normal levels.

## DISCUSSION

GMG is a chronic autoimmune disease that needs immunomodulatory drugs to achieve sufficient clinical improvement. Previous standard treatments included corticosteroids in combination with azathioprine or other immunosuppressants such as azathioprine, myco-phenolate mofetil, or tacrolimus [13, 14]. However, some patients with gMG do not fully respond to adequate dosing and sufficient treatment duration of these agents. The effectiveness of rituximab was increasing used in gMG [15]. The recent RINOMAX trial shows that early use of rituximab results in greater probability of minimal MG manifestations compared to placebo [16]. This suggests a feasible treatment algorithm in new-onset MG. Furthermore, rituximab also shows a greater benefit earlier in the disease course compared to conventional immunosuppressant therapy[17, 18]. However, the Beat-MG trial found that rituximab did not show a corticosteroid-sparing effect compared to placebo[19]. The challenge poses the need for new therapeutic strategies. Advances in immunology and translational development have seen the emergence of promising biological agents with good efficacy and safety.

MG is usually caused by pathogenic autoantibodies, with AChR(+) MG most occurring among patients. Previous studies show that IL-6 and its related signaling pathway are involved in the pathogenesis of MG. Sera IL-6was found to be correlated with the disease activity and severity in AChR(+) MG. IL-6 may promote the activation of Thelper 17 (Th17), T follicular helper (Tfh)[20], as well as B cell maturation and antibody production. Blockade of IL-6 signaling with anti-IL-6 antibodies suppresses experimented autoimmune MG (EAMG), indicating IL-6 may be a potential target for modulation of autoimmune responses in MG[20]. In EAMG, IL-6 mRNA expression in sections of seriated muscle was significantly increased and showed immune response in muscular tissue[21]. IL-6 was also found to promote muscle wasting by triggering a shift of the slow-twitch fiber to a more sensitive fast fiber phenotype[22]. From these studies, we postulated that blockade of IL-6 signaling may play dual roles in MG, not only by inhibiting peripheral humoral immune response, but by improving muscular power. A placebo-controlled randomized trial to evaluate the effect of tocilizumab in early-onset MG is warranted.

In this study, we found that tocilizumab started to reduce QMG and MG-ADL scores at 4 weeks after treatment initiation and resulted in a rapid improvement in muscle weakness, especially after three cycles of tocilizumab infusion at week 12. This beneficial effect was able to maintain to week 24. However, QMG and MG-ADL scores did not seem to show further improvement after week 24. There were no significant differences in either QMG or MG-ADL scores at week 48, compared to week 12. Our data demonstrated that the maximal clinical effects of tocilizumab occurred by 24 weeks and all patients remained stable thereafter. Different from the change of QMG and MG-ADL from week 24 to week 48, sera AChR-ab titers also decreased after 24 weeks in the follow-up. At week 48, we found negative AChR-ab in two patients and most patients still had detectable AChR-ab. Long-term maintenance therapy with tocilizumab may have the effect of continuously decreasing AChR-ab antibody titers. However, the correlation of sera AChR-ab titers with clinical severity warrants further study in a large population.

Drug-related adverse events were recorded in this study, and no serious adverse events were observed. The safety profile of tocilizumab rendered satisfaction for most patients.

This study has several limitations. It was a prospective study involving only a small sample size. The lack of a control group gave rise to another potential for bias. Besides, patients with impending crises and MuSK-ab (+) were excluded from the study. A larger sample and double-blind controlled trials are needed to further verify the effectiveness and safety of IL-6R inhibitors. Actually, two randomized controlled studies (Satralizumab, NCT04963270; tocilizumab, NCT05067348) are going on to explore the efficacy and safety of IL-6R blocking agents.

### Conclusions

This prospective study shows that tocilizumab may be an alternative to achieve a good clinical response in AChR (+) gMG.

### Ethics approval and consent to participate

This study was approved by the Ethics Committee of Tianjin Medical University, and all patients provided written informed consent.

All the methods used in the study were in accordance with the Declaration of Helsinki.
